# Risk Perceptions and Acceptability of Genetically Modified Mosquitoes for Malaria Control in Mali, 2025

**DOI:** 10.12688/openresafrica.16627.1

**Published:** 2026-05-29

**Authors:** Samba Diarra, Abou Sogodogo, Sarah Hartley, Seydou Doumbia

**Affiliations:** 1 Department of Education and Research in Public Health and Specialties (DERSP), University of Sciences Techniques and Technologies of Bamako Faculty of Medicine Odontostomatology, Bamako, Bamako Capital District, Mali; 2 University Clinical Research Center, UCRC, Bamako1, Bamako Capital District, 1805, Mali; 3 Business School, University of Exeter Department of Computer Science, Exeter, England, UK; 4 University Clinical Research Center, University of Sciences Techniques and Technologies of Bamako Faculty of Medicine Odontostomatology, Bamako, Bamako Capital District, 1805, Mali

**Keywords:** Risk perceptions, knowledge acceptance, genetically modified mosquitoes, malaria, Mali

## Abstract

**Introduction:**

Malaria remains a major public health issue in sub-Saharan Africa, especially in Mali. Despite the promising prospects of genetically modified mosquitoes (GMMs), their use raises several concerns. Community engagement is essential to the success of this tool. We assessed the Malian population’s knowledge and perception of the risks associated with this technology.

**Methods:**

The study was conducted in rural areas in the Koulikoro region and Bamako (urban). The survey included 874 heads of household. The statistical analysis was done on R 4.3.0. Univariable and multivariable logistic regression were performed to identify key factors associated with the risk perceptions of GMMs. The study was approved by the ethics committee of the University of Sciences, Techniques, and Technologies of Bamako. Written informed consent was obtained from each participant.

**Results:**

The median age was 42 years (IQR: 33, 54), and 20.0% had no formal education. Only 18.2% (159/874) had previously heard information about GMMs, mainly by health workers (61.6%) and radio broadcasts (20.8%). The overall rate of risk perception regarding GMMs was 47.7% (417/874), with the most cited concern being the transmission of unknown diseases (95.9%). A high level of education was positively associated with higher risk perception. As well as being unaware of GMMs, doubts about the efficacy and safety of GMMs. The acceptability rate of GMMs was 77%, conditional on assurances that technology would not cause harmful effects and would first be tested in a controlled, restricted environment before being deployed on a large scale.

**Conclusion:**

Robust community engagement is essential for both research and real-world implementation of GMMs. A Thorough understanding of the conditions related to acceptance of this technology offers a strategic pathway for researchers and policymakers to design GMM that is socially responsive and contextually appropriate.

## Introduction

Malaria remains the major public health issue in sub-Saharan Africa. Despite efforts, there were 282 million malaria cases globally in 2024, an increase of about 9 million cases (3%) compared to 2023. ^1^ In Mali, malaria is the leading cause of morbidity and mortality, accounting for approximately 3% (8 million) of global malaria cases and 2.4% (14,328) of global malaria deaths in 2024. ^1^ Although significant progress through the scale-up of long-lasting insecticide-treated nets (LLNs), seasonal malaria chemoprevention (SMC), rapid diagnostic testing, and artemisinin-based combination therapy (ACTs), progress toward malaria elimination is threatened by several persistent and emergent challenges. ^1^ These include increasing among vectors to insecticide, and emerging resistance to antimalarial drugs, ^2– 3^ weaknesses in the health system capacity, ^4^ climate variability influencing transmission dynamics, ^5^ and regional insecurity, ^6^ that disrupts service delivery and community engagement. ^1^

These persistent obstacles underscore the urgent need for innovative tools to sustain malaria control and progress toward elimination. ^7^ Emerging vector control strategies, such as genetically modified mosquitoes (GMMs), offer a potential transformative approach to improve the prospects for malaria elimination. ^8, 9^ Through advanced gene mechanisms, GMMs can either suppress mosquito populations or interrupt the parasite transmission, thereby complementing existing interventions and addressing their limitations. ^10, 11^ Similar approaches have been used to genetically modified *Aedes aegypti* mosquitoes, a species that transmits viruses such as Zika, dengue, and chikungunya. ^12– 14^ Experimental trials of such technology had already been implemented in other parts of the world, releasing male *A. aegypti* mosquitoes containing the self-limiting gene and monitoring species abundance. In Brazil, a reduction of 95% in A. aegypti populations over 6 weeks had been reported. ^12^ Similar, in Grand Cayman, a reduction of 82% in *A. aegypti* mosquitoes had been reported. ^13^

However, the use of genetically modified mosquitoes raises complex ethical, biosafety, regulatory, and governance concerns. ^15– 17^ Importantly, global debates on GMMs are largely dominated by actors in America and Europe and have rarely centered on African perspectives, despite Africa bearing the highest burden of malaria. ^18– 19^ Experiences from settings illustrate the social and political sensitivity surrounding genetically modified insects. In Florida, a proposed field trial of genetically modified *Aesdes aegyptis* mosquitoes to prevent Zika virus transmission triggered intense public controversy. ^20^ In Key Haven, signs reading “No consent” were displayed in approximately half of residential gardens. ^20^

In Africa, Target Malaria, an international research consortium established in 2012, aims to develop genetically modified mosquitoes (GMMs) for malaria control and has operated in Mali and Burkina, and currently in Uganda and Ghana. ^21^ In 2019, the Burkina Faso team conducted the first release of genetically modified sterile male mosquitoes on the African continent, although these mosquitoes did not carry a gene drive. While the release was scientifically limited and carefully regulated, it generated significant public debate. Civil society organizations, particularly COPAGEN (Coalition pour la Protection du Patrimoine Génétique Africain), have consistently opposed GMM research, arguing that “Burkina Faso, and Africa by extension, must not be considered a laboratory, and African populations must not serve as guinea gigs”. ^16^ In 2025, the Burkinabè government suspended Target Malaria’s activities, citing “national interest” as justification, fueling the speculation regarding community opposition, political pressures, sovereignty concerns, and biosecurity risks. ^22^

In Mali, activities conducted under Target Malaria between 2012 and 2023 focused primarily on entomological studies, including the importation and containment-based study of non-gene drive genetically modified sterile male mosquitoes. ^23^ While awareness-raising efforts have targeted the scientist and policies-makers, community engagement has been limited to small, homogenous local populations directly involved in field activities. ^23^ Broader public engagement has remained limited. ^24– 25^

These experiences demonstrate that scientific approval and regulatory clearance alone are insufficient to ensure sustainability. Social acceptability is decisive. For the successful design and implementation of GMM technology, community engagement is widely recognized as a critical pathway to achieving public acceptance. The growing emphasis on community engagement and people-centered approaches in global health, as promoted by the World Health Organization’s Framework for Integrated, People-Centered Health Services, highlights the importance of local participation in strategies development and policy formulation. It is therefore essential to assess the local communities’ awareness and understand how they perceive potential risks and benefits. In response to international calls for responsible research and innovation , ^26– 27^ this study aims to examine public understanding, attitudes, and perceptions of genetically modified mosquitoes in Mali.

## Methods and materials

### Study settings

**Figure 1.**
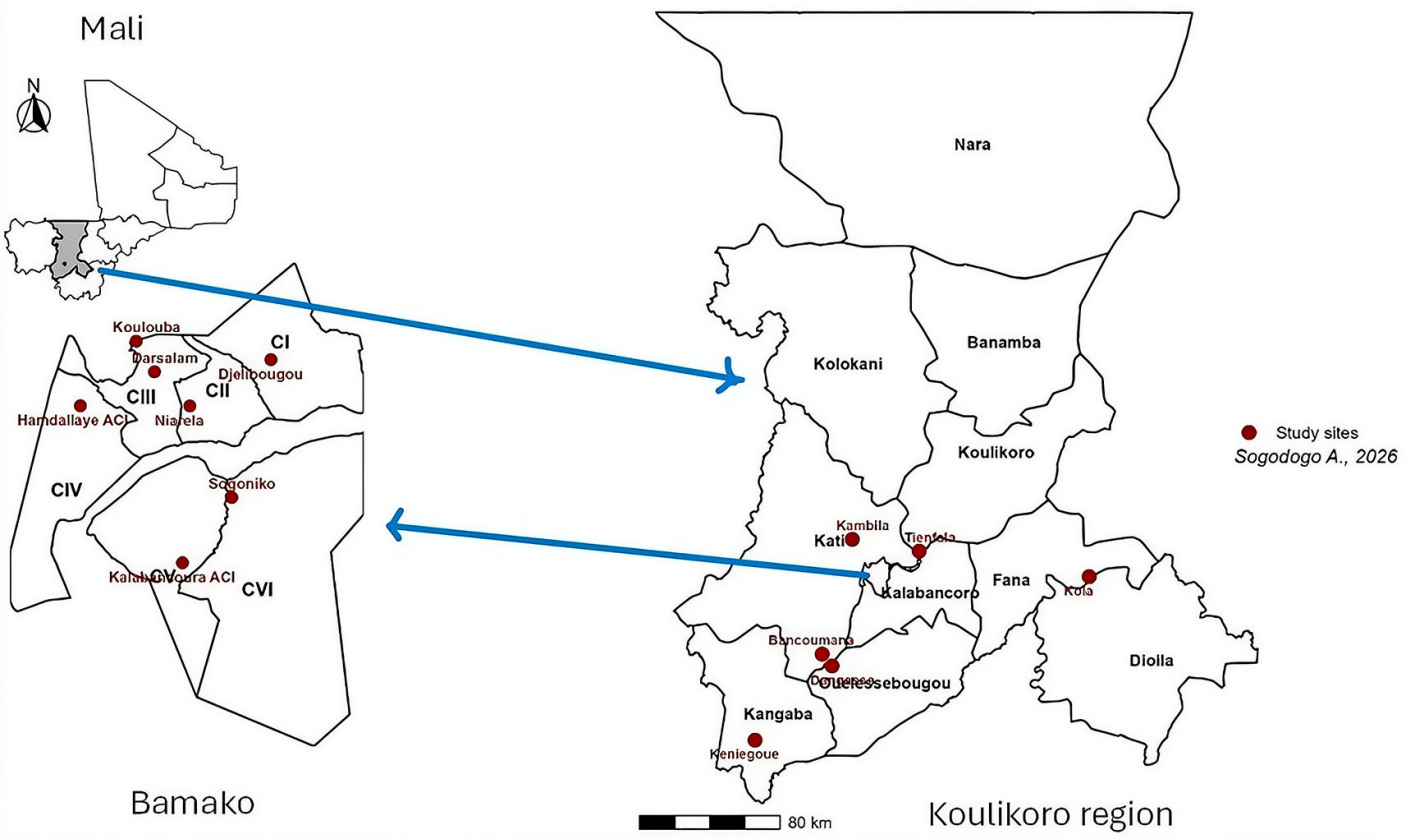
Geographic distribution of study sites in rural Koulikoro region (right) and urban Bamako (bottom left), Mali.

The study was done in the Koulikoro region (rural) and Bamako (urban). The Koulikoro region was selected because of the availability of historical data on malaria research activities. Its population is estimated at 4,737,164 inhabitants, and its demographic weight is 21.2%, the highest in the country (RGPH5, 2022). The Koulikoro region has extensive experience in malaria research. It has hosted several malaria research programs such as ICEMR, IVCC, and Target Malaria. It has also undergone indoor residual spraying campaigns with PMI, particularly from 2008 to 2016. The Koulikoro region is a prime malaria-endemic area. Bamako was chosen because of its status as an urban area and the presence of research and regulatory institutions involved in malaria control. Its population is estimated at 2,899,672 inhabitants, with a demographic weight of 12.9% (ibid). Like Koulikoro, Bamako is also an endemic malaria zone. The study involved heads of households in rural and urban communities. Were included in rural settings villages (Bancoumana, Dangassa, Kambila, Keniegoué, Kola, and Tienfala). Among them are those covered by the Target Malaria project ( Figure 1).

### Study population and sampling

The study population consisted of: (1) men or women aged 18 years or older who were household heads, (2) had lived in the study area for at least six months, and (3) were responsible for household decision-making.

### Sample size

The sample size for this study was calculated using Raosoft® http://www.raosoft.com/. According to Mali’s fifth general population and housing census, the populations of Bamako and Koulikoro are estimated at 2,899,672 and 4,737,164 inhabitants, respectively (RGPH5, 2022). Using a 95% confidence level, a 5% margin of error, and a response distribution of 50%, the minimum required sample size was calculated to be 770 household heads across both sites (385 per site). To account for potential non-response and incomplete questionnaires, we increased the sample size by 10%, resulting in a final target sample of 847 participants. A minimum of 70 was required by the village or neighborhood.

### Recruitment procedure

Within each selected village or neighborhood, the survey began at the household of the village chief. The first household was randomly selected by splining a pen on the ground and following the direction indicated by its tip. From that starting point, the interviewers visited consecutive households until the required number of participants for that locality was reached. For the survey, a household was defined as a group of people who lived together and shared meals from the same cooking pot. In a concession where multiple households resided, only one household was randomly selected.

### Study design and period

We conducted a quantitative cross-sectional study among heads of household in the community population in Mali. The study was conducted between August and November 2025.

### Data collection

A household survey was conducted by three trained interviewers. The questionnaire was designed on Kobocollect with conditions applied to facilitate interviews and then deployed on tablets. Before the actual survey, a pretest was conducted on a sample of 23 heads of households to ensure that the data collection tools were well adapted. The questions focused on the heads of households’ knowledge of malaria and genetically modified mosquitoes, their experience with vector control methods, their perceptions of the risks associated with genetic modification, and the risk management mechanisms proposed for genetic modification. They were also asked about their future acceptance of the implementation of GMMs and their proposals for effective research and implementation of this intervention.

### Data analysis

We used R software version 4.3.0 to analyze the data. Descriptive statistics were used to summarize the variables. Univariate and multivariate logistic regressions were performed to identify factors associated with the perception of risks associated with the use of genetically modified mosquitoes. Age variables were categorized into age groups. For the multivariate analysis, we performed stepwise backward logistic regression to determine the best model with the lowest AIC. Crude and adjusted odds ratios were presented with the 95% confidence interval. To explore the conditions influencing public acceptance of the GMMs, we conducted a word cloud analysis using R. This visualization technique allows us to summarize the most prominent themes extracted from the relevant textual data.

### Ethical considerations and approval

The study was approved by the ethics committee of University of Sciences Techniques and Technologies of Bamako under N°2024/193/CE/USTTB. Written informed consent was obtained from each participant prior to their involvement in the study. Surveys were conducted in quiet and secluded locations to ensure confidentiality and foster a focused and comfortable environment for participants. To ensure anonymity, no personal identifiable information was collected for anonymity at any stage of the research process.

## Results

### Description of study participants

In total, 874 participants were surveyed. 51% of participants were surveyed in the rural areas. The number of participants in each village/municipality was between 70 and 80. More than 98% were married and 43% were aged between 20 and 40 years. 19.9% were unschooled, while 32.4% had primary school level. The sociodemographic characteristics are summarized in Table 1.

**Table 1.** Study information and sociodemographic characteristics of participants, 2025.

Characteristics	n (%)
**Zone**	
Rural	446 (51.0)
Urbain	428 (49.0)
**Marital**	
Married	859 (98.3)
Widower/divorced	15 (1.7)
**Categories of age (year)**	
18–20	4 (0.5)
20–40	377 (43.1)
40–60	345 (39.5)
≥ 60	148 (16.9)
Median of age (Q1, Q3) ^1^	42(33, 54)
**Schooling**	
Alphabetized/Self-taught	38 (4.3)
Madrasa/Coran school	119 (13.6)
Unschooled	174 (19.9)
Primary school	283 (32.4)
Secondary school	140 (16.0)
High school	120 (13.7)

n: the number of respondents to the questionnaire in households;

(%): percentage of respondents to the questionnaire in households

^1^ Q1 & Q3 = First and third quartiles.

### Awareness and knowledge of malaria

Knowledge and Perceptions of Malaria among Household heads on Genetically Modified Mosquitoes in Mali are presented in Table 2. Among the 874 participants, 57% had seen or heard information about malaria control in the last three months, mainly by radio (69%) and television (47%). Fever and vomiting were the main symptoms cited by 73.8% and 62.9% of participants, respectively. Almost all perceived malaria as a public threat in their community (96.2%), and 78.6% of them had reported at least one case of malaria in their families in the three previous months. Mosquito bites were known as the main means of malaria transmission by 85%, and insecticide-treated mosquito nets were the most cited intervention.

**Table 2.** Knowledge and Perceptions of Malaria among Household heads on Genetically Modified Mosquitoes in Mali, 2025.

Characteristics	n (%)
Heard information on fight against malaria (n = 874)	
No	376 (43.0)
Yes	498 (57.0)
Sources of information (n = 498)	
Radio	344 (69.0)
Television	236 (47.0)
Health workers and researchers	150 (30.0)
Village health volunteers	57 (11.0)
Relatives	40 (8.0)
Internets/messages	21 (4.2)
Knowledge of malaria symptoms/signs	
Fever	645 (73.8)
Vomiting	550 (62.9)
Muscle aches	434 (49.7)
Headaches	432 (49.4)
Diarrhea	131 (15.0)
Stomachaches	163 (18.6)
Dizziness	48 (5.5)
Tiredness	33 (3.8)
Nausea	25 (2.9)
Yellowing of the eyes	17 (1.9)
Anorexia	13 (1.5)
Perceived malaria as public health problem (n = 874)	
Yes	841 (96.2)
No	33 (3.8)
At least one case reported in last three month in the family	
No	187 (21.4)
Yes	687 (78.6)
Knowledge of malaria transmission methods	
Mosquito bites	746 (85.4)
Lack of hygiene	81 (9.2)
Consumption of oil and eggs	25 (2.9)
Other *	15 (1.7)
Interventions of malaria control	
Insecticide-treated mosquito nets	741 (84.8)
Antimalarial drugs	531 (60.8)
Traditional plants/decoctions	331 (37.9)
Sanitation	327 (37.4)
Insecticides, creams, repellents, and coils	211 (24.1)
Vaccination	80 (9.2)
Indoor spraying	49 (5.6)
Genetic modification of mosquitoes	19 (2.2)

n: the number

(%): percentage

* Freshness/rainfall (5), consumption of mangoes/sweet fruits (4), climate change (2), work-related fatigue (2), natural illness (1), poverty (1)

### Awareness and knowledge of genetically modified mosquitoes

Awareness and Knowledge of Household Heads on Genetically Modified Mosquitoes for Malaria Control in Mali are summarized in Table 3. Overall, 10.8% of participants were aware of these mosquitoes, and 18% had seen or heard messages mainly through health workers/researchers. Among those who were aware of GMMs, 28% were aware of activities in Mali. Regarding legislation, 12% were aware of the law on genetically modified organisms in Mali.

**Table 3.** Awareness and knowledge of household heads on genetically modified mosquitoes for malaria control in Mali, 2025.

Characteristics	n (%)
Aware of genetically modified mosquitoes (n = 874)	
No	780 (89.2)
Yes	94 (10.8)
Head/saw information on GMMs (n = 874)	
No	715 (81.8)
Yes	159 (18.2)
Sources of information on GMMs (n = 159)	
Health workers, and researchers	98 (61.6)
Radio	33 (20.8)
Internets/messages	20 (12.6)
Relatives	18 (11.3)
Television	12 (7.5)
Poster	2 (1.2)
Awareness of GMMs activities in Mali (n = 159)	
No	356 (71.5)
Yes	142 (28.5)
Awareness of legislation on GM organisms in Mali (n = 874)	
No	767 (87.8)
Yes	107 (12.2)

### Perceived benefits and management of GMMs

The perceived benefits and management of GMMs are reported in Table 4. In the study, 88.4% of participants had an excellent/good feeling about the introduction of genetically modified mosquitoes. Most participants believed that these mosquitoes could help combat the disease (72%) or reduce its burden (69%). In terms of confidence and capability, 62% said they were convinced that Malians could prevent potential side effects or manage GMMs properly. However, only 36% of respondents believed that this technology was safe.

**Table 4.** Perception of household heads regarding the benefits and management of genetically modified mosquitoes for malaria control in mali, 2025 (N = 874).

Characteristics	n (%)
Feelings on GMMs’ introduction into malaria interventions	
Excellent/good	773 (88.4)
Bad	61 (7.0)
Don’t know	40 (4.6)
Believe that GMMs will help combat malaria	
Yes	628 (71.9)
No	246 (28.1)
Belief in the efficacy of GMMs to reduce malaria	
Yes	605 (69.2)
No	269 (30.8)
Trust in Mali’s scientists/government’s ability to manage the GMMs	
Yes	542 (62.0)
No	332 (38.0)
Trust in Mali’ scientist/government to prevent the GMMs’ effects	
Yes	548 (62.7)
No	326 (37.3)
Believing in the full safety of GMMs	
Yes	317 (36.3)
No	557 (63.7)

### Perceptions of risks related to the use of GMMs in malaria control in Mali.

In terms of perceived risk associated with the use of GMMs, 47.7% reported perceiving risks related to their use. The main risks were the transmission of unknown diseases (96%). These results are reported in Table 5.

**Table 5.** Perceptions of risks associated with the use of genetically modified mosquitoes in malaria control among household heads in mali, 2025.

Characteristics	n (%)
**Perception of risk on GMMs use in malaria control (n = 874)**	
Yes	417 (47.7)
No	457 (52.3)
**Types of risk on GMMs use in malaria control (n = 417)**	
Risk of unknown diseases	400 (95.9)
Risk of damage to ethical values	195 (46.8)
Risk of damage to human health	161 (38.6)
Risk of damage to environmental health	150 (36.0)
Risk of damage to animal health	149 (35.7)

### Community acceptance of GMMs for malaria control in Mali, 2025 (N = 847)

**Figure 2.**
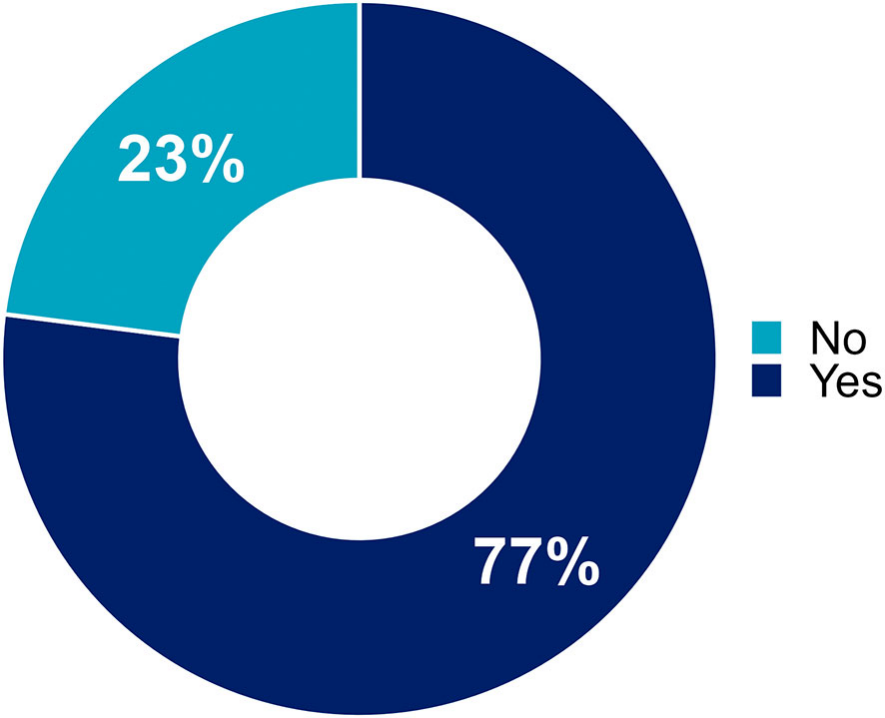
Acceptability of genetically modified mosquitoes for malaria control among household heads in mali, 2025.

Acceptability of Genetically Modified Mosquitoes and Conditions among household heads in Mali are reported in Figure 2. The community acceptance rate of genetically modified mosquitoes for malaria control was 77%. However, this acceptability was conditional on the certainty that they would not have any unforeseen consequences and that the technology would be tested in a controlled and limited environment before being deployed on a larger scale.

### Factors associated with the risk perceptions of genetically modified mosquitoes

Sociodemographic and Contextual Factors Associated with Risks Perceptions of GMMs are presented in Table 6. Risk perception regarding the genetically modified mosquitoes was significantly three times higher among participants with a high school education compared to those without formal schooling. Individuals who were unaware of GMMs before the study were also more likely to perceive technology as risky. Key drivers of these perceptions included doubts about the efficacy and safety of GMMs.

**Table 6.** Sociodemographic and contextual factors associated with risks perceptions of genetically modified mosquitoes in malaria control among household heads in mali, 2025.

	OR (95%IC)	*p*	aOR (95%IC)	*p*
Zone				
Rural	–	–	–	–
Urban	1.44 (1.10–1.88)	0.01	1.05 (0.73–1.49)	0.80
Age (year)	0.99 (0.98–1.00)	0.05	0.99 (0.98–1.00)	0.19
Schooling				
Unschooled	–	–	–	–
Madrasa/Coran school	1.26 (0.79–2.02)	0.34	0.93 (0.40–2.22)	0.86
Alphabetized/Self-taught	0.83 (0.39–1.69)	0.61	1.24 (0.52–3.08)	0.63
Primary school	1.19 (0.81–1.74)	0.38	1.52 (0.67–3.59)	0.33
Secondary school	1.73 (1.11–2.72)	0.02	1.95 (0.81–4.84)	0.14
High school	1.92 (1.20–3.08)	0.01	2.91 (1.17–7.51)	0.02
Marital status				
Married	–	–		
Widower/divorced	0.96 (0.33–2.69)	0.93		
Heard/see information on the fight against malaria				
Yes	–	–	–	–
No	1.71 (1.31–2.25)	<0.01	1.31 (0.94–1.82)	0.11
Perceived malaria as public health issue				
Yes	–	–	–	–
No	2.26 (1.10–4.89)	0.03	1.05 (0.44–2.58)	0.91
At least one malaria case reported in last three months in the family				
Yes	–	–	–	–
No	1.98 (1.43–2.76)	<0.01	1.43 (0.97–2.12)	0.08
Aware of malaria transmission means				
Good	–	–		
Bat	1.24 (0.85–1.81)	0.26		
Aware of GMMs				
Yes	–	–		
No	1.20 (0.78–1.86)	0.40		
Heard/saw information on GMMs				
Yes	–	–	–	–
No	1.59 (1.12–2.27)	0.01	1.54 (1.01–2.37)	0.05 *
Aware of legislation on GMOs in Mali				
No	–	–		
Yes	0.74 (0.49–1.11)	0.15	0.67 (0.42–1.08)	0.10
Feeling on GMMs introduction into malaria interventions				
Excellent/good	–	–	–	–
Bad	3.51 (1.99–6.50)	<0.01	1.09 (0.53–2.29)	0.82
Trust in Mali’ scientists/government ability to manage the GMMs				
Yes	–	–	–	–
No	1.75 (1.33–2.31)	<0.01	0.84 (0.57–1.23)	0.38
Trust in Mali’ scientist/government to prevent the GMMs’ effects				
Yes	–	–	–	–
No	1.68 (1.28–2.22)	<0.01	0.76 (0.51–1.13)	0.18
Belief of the efficacy of GMMs to reduce malaria				
Yes	–	–	–	–
No	2.93 (2.17–3.96)	<0.01	2.10 (1.00–4.49)	0.05 *
Believing in the safety of GMMs				
Yes	–	–	–	–
No	4.03 (2.99–5.46)	<0.01	3.20 (2.23–4.61)	<0.01 *
Believe that GMMs will help combating malaria				
Yes	–	–	–	–
No	2.89 (2.13–3.95)	<0.01	0.77 (0.34–1.70)	0.52
Accept the use of GMMs for malaria control				
Yes	–	–	–	–
No	4.62 (3.26–6.64)	<0.01	3.94 (2.41–6.57)	<0.01 *

**OR (IC95%)**: Crude odds ratio with a 95% confidence interval;

**p**: p-value

**a; OR (IC95%)**: Adjusted odds ratio with a 95% confidence interval;

**p**: post-adjustment p-value

* Significance factors associated

## Discussion

This study involved an important number of local community leaders in rural and urban areas, which is very crucial regarding the discussion around genetically modified organisms. The potential public health impact of genetically modified mosquitoes requires strong community engagement at all stages of development. This study is among the first to include diverse populations from rural and urban communities, as well as villagers both involved and not involved in malaria research. It provides a comprehensive overview of the general population’s knowledge of GMMs, offering critical evidence to inform and tailor future community engagement strategies.

### Knowledge and perceptions of household heads on malaria

Participants demonstrated high awareness of malaria, largely shaped by radio and television messaging. These findings likely reflect sustained national communication efforts, particularly intensified during high-transmission seasons, when community and political leaders are mobilized to support dissemination activities. Messages are strategically delivered through targeted channels such as local radio, television and public engagement events conducted alongside mass interventions campaigns and distribution activities. Similar patterns have been reported by Okafor et al ^28^ in Uganda, Aragie et al ^29^ in Ethiopia, and Mathania et al ^30^ in Tanzania, where mass media and health workers were identified as primary sources of information.

Malaria was widely recognized as a major public health problem (96.2%), reflecting lived experience, frequent household exposure, and the country’s high disease burden. Most participants (>75%) reported at least malaria cases within their family. Fever and vomiting were commonly identified symptoms, and 85.2% correctly cited mosquitoes as the mode of transmission. These results consistent with findings from other African settings. ^28, 30, 31^ High knowledge of transmission and symptoms is critical for prevention and timely care-seeking.

However, important misconceptions persisted, including beliefs that malaria results from certain foods (oils, eggs, or mangoes/sweet fruits), poor personal hygiene, and as a “natural” disease unrelated to mosquito bites. Similar misconceptions have been documented in Ethiopia by Zegene et al ^32^ were participants attributed malaria transmission to sleeping near malaria patients, contact with an infected person’s sweat, poor personal hygiene, hunger, and other insect bites. Such misunderstandings have also been widely reported across African countries. ^33– 34^ Such misunderstandings may delay appropriate care and undermine prevention efforts. While insecticide-treated nets were the most frequently cited preventive measure, targeted communication strategies remain necessary to address persistent myths and strengthen an accurate understanding of malaria transmission and control.

### Awareness and knowledge of genetically modified mosquitoes among household heads

This study highlighted limited awareness of genetically modified mosquitoes within communities, with health workers and researchers constituting the principal sources of information. Similar findings have been reported in Ghana, where a survey conducted at the University of Ghana and surrounding communities found that 63.6% of respondents had never heard of GMMs. ^35^ By contrast, a study in Nigeria documented higher levels of knowledge among scientists, 60% of whom reported having heard or read about GMMs, primarily through internet sources and scientific publications. ^36^ Likewise, a multi-country study involving 180 researchers and malaria control program members across 25 sub-Saharan African countries found that 94.3% of participants were aware of GMMs, mainly through scientific articles, scientific meetings and conferences, friends/colleagues, and social media, yet only 28.3% reported understanding how GMMs function in malaria control. ^37^

These results reveal a significant communication gap surrounding GMMs research in African settings. Awareness remains limited among community members and incomplete even among some malaria stakeholders. Such a gap risk undermining public trust, informed decision-making, and the social legitimacy of vector emerging control interventions. Strengthening community engagement is therefore imperative. Engagement strategies should be accessible, sustained, and culturally grounded, emphasizing clear communication, iterative dialogue, and the involvement of trusted local actors. These approaches are essential to promote informed participation and to uphold ethical and regulatory standards. Evidence suggests that multiple communication modalities can be mobilized effectively in sub-Saharan Africa. Bunn et al. ^38^ identified 17 different categories of art forms commonly used for public engagement, with theatre, music and song, television and radio, visual are and storytelling among the most prevalent. In this context, Mananan et al., ^39^ have demonstrated that culturally embedded songs can enhance understanding and foster acceptance of GMMs. in promoting community engagement and acceptance of GMMs. Communication about GMMs and community engagement should consider the context to be more effective and acceptable.

Regulatory preparedness further reinforces the need for informed public dialogue. Many African countries are developing a biosafety framework governing genetically modified organisms (GMOs). In Mali, Law No. 08–042/AN-RM relating to safety in Biotechnology in the Republic of Mali was stolen from the National Assembly on December 1, 2008, regulates the import, export, transit, contained use, release, or placing on the market of GMOs, whether intended for environmental release, food, feed, pharmaceutical use, or a processed products delivery from GMOs. The law also covers GMOs with a dual agricultural and pharmaceutical. ^40^ This legislative framework provides a formal basis for oversight of GMM research and potential deployment.

However, regulatory implementation often requires evidence of prior consultation and public acceptance. In our study, awareness of this national biosafety law was strikingly limited: among participants who had heard of GMMs, only 12.2% knowledge of the existing GMOs law. This gap between regulatory architecture and public awareness may weaken the legitimacy of decision-making processes and limit meaningful participation. Transparent dialogue is therefore essential not only to build trust but also to facilitate compliance with the national biosafety authorities and align with international guidance. ^41^

A decade-long (2012–2023) review of Target Malaria activities in Mali highlights that early, sustained, and culturally appropriate dialogue, incorporating iterative communication, local partnerships, adaptive consent, and structured feedback, is essential for building trust, improving understanding of gene drive technologies, and integrating community perspectives. Experiences in Uganda and other African countries confirm that engagement must be ongoing, context-specific, and responsive to local concerns to ensure the ethical and socially responsible development of genetically modified mosquitoes. However, 28% of our participants reported being aware of GMM research activities in Mali. These results demonstrate a need to adapt communication strategies for stronger, more accessible, and more sustainable community engagement strategies.

### Perceptions of household heads on genetically modified mosquitoes

Most participants (88.4%) expressed a positive perception of the potential introduction of GMMs for malaria control and believed that GMMs could effectively held combating and reducing malaria burden. Our results are consistent with Finda et al., ^37^ in which 78.3% of participants reported optimism about GMMs, citing advantages such as effectiveness, sustainability, affordability, and feasibility. In Nigeria, 56.1% of scientists also considered GMMs useful for malaria control. ^36^ These results demonstrate a generally favorable climate for the implementation of GMMs, both at the community and scientific levels.

However, as in most publications, participants expressed strong concern about GMMs (47.7%), mainly the risk of unknown diseases. Other risks were cited, such as risks of damage to human health, environmental and animal health. In our study, risk perception regarding genetically modified mosquitoes was significantly higher (three times greater) among participants with a high school education compared to those without formal schooling. Individuals who were unaware of GMMs prior to the study were also more likely to perceive the technology as risky. Key drivers of these perceptions included doubts about the efficacy and safety of GMMs, highlighting the importance of targeted education and transparent communication to address public concerns and foster informed acceptance.

Similar findings have been reported in Mali in 2010, which documented that while many community members were open to GMM technology, reservations were expressed about human health and environmental consequences. ^42^ According to a study conducted among civil society groups in Tanzania, ^43^ concerns were raised about the operation and long-term ecological effects of GMMs, the lack of clarity regarding responsibility and accountability, limited transparency in research, insufficient local scientific capacity, transboundary impacts, growing technological dependence, and the marginalization of local solutions. In Nigeria, ^36^ some have expressed concerns about the risk of these mosquitoes spreading beyond their release sites or hybridizing with other mosquito species. In Ghana, the concerns listed among university members and the surrounding community were danger to humans and the environment, resistance of GMMs to control measures, transmission of other diseases, ethical issues, and effectiveness of the technology. ^35^ Marshall et al ^42^ have documented that the most frequently cited concern is that the GMM project “will no work”. These concerns reflect not only a lack of trust in scientific mechanisms but also a general apprehension about invisible and difficult-to-control risks, especially since GMMs remain little known and are perceived as coming from outside.

### Acceptability of genetically modified mosquitoes among household heads

More than three-quarters of participants said they would accept GMMs if they were sure that they had no side effects, they were tested in restricted areas and were overall safe. Similar findings had been made in previous studies in Mali, but also in the general literature. ^36, 37,43^ In Nigeria, ^36^ 83.5% of scientists expressed skepticism about the potential release of GMOs in their country, emphasizing the importance of emergency measures to eliminate these GMOs should any risks arise during their release. Finda et al., ^37^ have underscored the importance of generating context-specific, country-relevant evidence to assess the effectiveness and safety of GMMs. ^37^ These conditions included rigorous safety validation through locally conducted trials, transparent access to experimental results, and collaborative oversight involving foreign organizations, local experts, and international bodies to foster trust. In addition, equitable implementation, characterized by community engagement, infrastructure support, and the absence of financial burden, was also identified as a critical prerequisite for public acceptance and successful deployment of GMMs.

Although previous work suggested that public confidence depends heavily on the credibility of regulatory authorities and the perceived adequacy of oversight. Failures in governance or risk regulation can rapidly erode trust. These findings align whit the WHO policy framework, which mandates that communities must be meaningfully involved and granted the opportunity to give legitimate consent prior to any release of GMMs. The demand for clear evidence and accountability reflects a broader ethical imperative: innovation must be transparent, inclusive, and responsive to local realities. Sustained dialogue and participatory governance are therefore essential for GMMs research and implementation.

## Conclusion

Community knowledge of genetically modified mosquitoes remains limited, despite ongoing research activities. Nonetheless, perceptions of GMMs as a malaria control tool are generally positive. Concerns were more focused on potential unforeseen health effects, safety, and the efficacy of technology. Importantly, most respondents reported conditional acceptance of GMMs, contingent upon assurances that the technology would not cause harmful effects and would first be tested in a controlled, restricted environment before being deployed on a large scale. Inclusive, transparent, and sustained community participation is essential. By actively involving communities in decision-making and ensuring that their voices influence the development and deployment of GMMs, stakeholders can foster a shared sense of ownership and responsibility. This approach is essential to achieving ethical, effective, and socially acceptable control of malaria through genetically modified mosquitoes.

## Data Availability

The data used in this study are available from the corresponding author upon reasonable request. Data generated or analyzed during this research are stored in accordance with institutional requirements and applicable ethical guidelines. The questionnaire used in this study is available at Fisghare: https://doi.org/10.6084/m9.figshare.32252442. Data are available under the terms of the Creative Commons Attribution 4.0 International license (CC-BY 4.0). ^44^
