# Supplementary material for: Awareness, Risk Perceptions and Acceptability of Genetically Modified Mosquitoes for Malaria Control in Mali, 2025
Source: Open Res Afr. 2026 Jul 21;9:30. Originally published 2026 May 29. [Version 2] doi: 10.12688/openresafrica.16627.2 (PMC13310356; doi:10.12688/openresafrica.16627.2)
Supplement: Peer review history

# Reviewer report: Reviewer response for version 1

**DOI:** 10.21956/openresafrica.17819.r34920

**Published:** 2026-06-27

**Authors:** Neera Kapoor

**Affiliations:** 1 IGNOU, New Delhi, Delhi, India

Very interesting article.  Objectives are well formulated. Extensive Literature survey is done Sample size could have been large for appropriate statistical analysis. Results are supplemented by Tables but Table 6 seems to be a bit confusing. Risk perceptions to be elaborated. Introduction to some GM techniques could have been added. Discussion is well written. Concluded well. Some future line of investigation could have been added.

Is the work clearly and accurately presented and does it cite the current literature?

Yes

If applicable, is the statistical analysis and its interpretation appropriate?

Yes

Are all the source data underlying the results available to ensure full reproducibility?

Yes

Is the study design appropriate and is the work technically sound?

Yes

Are the conclusions drawn adequately supported by the results?

Yes

Are sufficient details of methods and analysis provided to allow replication by others?

Yes

Reviewer Expertise:

Mosquito ecology, ITN, mosquito control, artemisinin therapy, Plasmodium

I confirm that I have read this submission and believe that I have an appropriate level of expertise to confirm that it is of an acceptable scientific standard.

# Reviewer report: Reviewer response for version 1

**DOI:** 10.21956/openresafrica.17819.r34928

**Published:** 2026-06-27

**Authors:** Geoffrey R Hosack

**Affiliations:** 1 CSIRO, Tasmania, Australia

Review of “Risk Perceptions and Acceptability of Genetically Modified

 Mosquitoes for Malaria Control in Mali, 2025”

 This study provides insight into the important issue of community awareness and understanding of a novel biotechnology developed to improve public health. The study documents variability across communities in Mali with respect to how the potential benefits and risks of genetically modified mosquitoes developed to combat malaria are perceived. The survey documents how individuals in two regions of Mali are informed and educated on malaria and treatment strategies including the potential deployment of genetically modified mosquitoes. Recommendations are provided to increase communities’ participation and knowledge of genetically modified mosquitoes as a potential strategy for reducing the burden of malaria. The following specific comments identify areas in the paper that require clarification to aid readers’ understanding of the important contributions and conclusions of this study.

 Specific comments (square brackets [] in quotes mark suggested edits)

 Abstract, first paragraph, last sentence – rephrase to reflect restricted spatial scope of study in Mali: “We assessed knowledge and perceptions of the risk associated with this technology [in Mali].”

 Abstract, second paragraph – omit “The statistical analysis was done on R 4.3.0”. This information is helpful to include in Methods section but does not to be stated in the Abstract.

 Abstract, third paragraph – incomplete sentence: “As well as being unaware of GMMs, doubts about the efficacy and safety of GMMs.”  Were doubts stated by participants?

 Abstract, fourth paragraph – typo: “A [t]horough understanding …”

 Page 3, 1 ^st^ paragraph, last sentence – suggest rephrasing and splitting into two sentences: “These include increasing [mosquito vector resistance] to insectide[s] and emerging resistance … and regional insecurity[. These challenges disrupt] service delivery …”

 Page 3, 2 ^nd^ paragraph, third to last sentence – suggestion: “… releasing [non gene-drive, self-limiting genetically modified *A. aegypti* mosquitoes.]”

 Page 3, 2 ^nd^ paragraph, second to last sentence -- italicise “ *A. aegypti*”.

 Page 3, last paragraph, first sentence – much of this paragraph relates to geographic scope of study. I suggest moving up reference to figure 1 from last sentence of this paragraph to first sentence: “The study was done in the Koulikoro region (rural) and Bamako (urban) [, see Figure 1].”

 Page 4, 1 ^st^ paragraph -- here and elsewhere, citation for (RGPH5, 2022) should be numeric

 Page 4, 1 ^st^ paragraph -- spell out acronyms at first use: “President’s Malaria Initiative (PMI)”

 Page 4, 1 ^st^ paragraph, second to last sentence – rephrase, e.g., “The villages of Bancoumana, Dangassa, Kambila, Keniegoué, Kola, and Tienfala were included [as rural settings].”

 Page 4, 1 ^st^ paragraph, last sentence – reference to Target Malaria study sites: it’s unclear how this study relates to Target Malaria activities. For example, the legend for Figure 1 identified sites for the survey conducted in this study but does not refer to Target Malaria study sites. It will be important to add contextualisation about how this study relates to Target Malaria activities or add clarification on how the survey sites overlap with areas of active malaria research.

 Page 4, “Sample size” paragraph – report goal for the minimum required sample size calculation. What is being estimated for the statistical power analysis? Would it also be better to say that a response percentage (rather than distribution) of 50% was assumed for the purpose of the power analysis? It may also be more consistent to refer to two “areas” rather than “sites” here because Figure 1 shows sites as points within the two areas of Bamako and Koulikoro region.

 Page 4, “Recruitment procedure” paragraph – “survey began at the household of the village chief”: this statement appears to conflict with next sentence where the first household is said to have been randomly selected, please clarify.

 Page 5, “Data collection” paragraph – provide link or citation for “Kobocollect”

 Page 5, “Data analysis” paragraph – Both univariate and multivariable logistic regressions are described. Please provide enough detail about these different logistic regressions to allow interpretation of Table 6 in the Results. Are there many different regression results summarised together or one single multivariable logistic regression? Advise the reader of the difference between crude and adjusted odd ratios that both appear in Table 6. The “word cloud analysis” does not appear in Results and so can be removed from Methods.

 Table 1, 4 ^th^ row – typo: “Urbain” should be “Urban”

 Page 6, “Community acceptance of GMMs …” – Is it possible to describe or provide more information on the participants’ conditional acceptance, e.g., statements about investigation of potential unforeseen consequences or controlled and limited testing of GMMs before deployment? This could be an important contribution of the study. It might also help to describe or provide reference to questionnaire wording here that provides relevant background conditions to the survey responses.

 Page 6, “Factor associated with the risk perceptions” – many estimates are presented in Table 6 that are not mentioned here, perhaps more can be said about important results from this study in summary form?

 Page 6, Discussion, first paragraph – “villagers both involved and not involved in malaria research”. This variable was not explored in the presented analysis or results from the survey. Was the sample size too small for reliable estimates with respect to the possible effect of malaria research on perspective and awareness? Should the effect of malaria research on perspectives and awareness be further explored in future research?

 Table 4, row 15 –typos: “Trust in Mali’[s] scientist[s]/government to prevent [negative effects from GMMs]”?

 Page 10, second paragraph -- “Biotechnology in the Republic of Mali … regulates”: this sentence is confusing and revision is required to address including correction of any typographical errors or inadvertent mistranslation (e.g., translating volé instead of voté due to typographical error).

 Page 10, second paragraph – “The law also covers GMOs with a dual agricultural and pharmaceutical [use].”

 Page 10, third paragraph, second sentence – “… only 12.2% [had] knowledge …”

 Page 10, fourth paragraph – add citation for “review of Target Malaria activities in Mali”

 Page 10, last paragraph, first sentence – “… could effectively [combat and reduce] malaria burden.”

 Table 6 caption – Please provide guidance on interpretation of Table and how its results are grouped and partitioned.

 Table 6 first row – 0.95 confidence intervals are abbreviated as “CI”

 Table 6, row 25 – “Aware[ness] of malaria transmission [factors]”

 Table 6, row 26 – typo: “Bat” should be “Bad”

 Page 13, second paragraph, third sentence – “These findings align [with] the WHO policy framework …” please include the WHO reference.

 Data availability statement – It is stated that the data for this study are available on request. For the review question “are source data … available to ensure full reproducibility?”, I have responded with “partly”: The journal says “ *We require that all data underlying figures and tables are provided (unless there are issues of confidentiality) as part of the article or deposited in a public repository. The data should be described in a usable and accessible format.*” Please confirm that the study complies with the journal data availability policy. If unidentifiable data are not made available to help ensure confidentiality or comply with institutional data storage requirements, then perhaps this reason should be explicitly included in the availability statement.

Is the work clearly and accurately presented and does it cite the current literature?

Partly

If applicable, is the statistical analysis and its interpretation appropriate?

Partly

Are all the source data underlying the results available to ensure full reproducibility?

Partly

Is the study design appropriate and is the work technically sound?

Yes

Are the conclusions drawn adequately supported by the results?

Partly

Are sufficient details of methods and analysis provided to allow replication by others?

Partly

Reviewer Expertise:

Risk analysis

I confirm that I have read this submission and believe that I have an appropriate level of expertise to confirm that it is of an acceptable scientific standard, however I have significant reservations, as outlined above.

# Reviewer report: Reviewer response for version 1

**DOI:** 10.21956/openresafrica.17819.r34801

**Published:** 2026-06-18

**Authors:** Marceline F. Finda

**Affiliations:** 1 Ifakara Health Institute, Ifakara, Tanzania

**Overall comment**

 Great article, it aligns well with other work that has been done across Africa. Below are a few edits to the specific sections:

**Abstract**

 In the results section, characteristics of the participants are not the core of your study; you do not need to include this info in the abstract, just go straight to the point. 

 In the conclusion section, you need to clarify that the acceptance was conditional on proof of safety; that is an important finding. 

 Correction: 'Thorough' should be lowercase.

**Introduction**

 Is this particular study funded by Target Malaria? I see that you have dedicated two paragraphs to explaining TM work in Mali and Burkina, which I think is a bit too much; I would suggest summarizing this. If not funded by TM, then that is even more controversial, as you are focusing on only TM work, despite other groups working on GM work across Africa. 

**Methods**

 This section is clearly articulated. My concern here is on how these concepts were defined and assessed. How did you determine that the participants understood what you meant? How did you define GMM, risks, concerns, etc.? Especially in rural settings, where participants may not fully understand the Western languages? Different GMM technologies pose different concerns. How did you determine which technologies respondents were referring to, or did you differentiate them? Did you provide any information to the respondents before asking them questions? All of this info needs to be included in the methods section. 

**Results**

 It is always good to include the n-value after each percentage you present to indicate the context; for example, 51% (n = 445) were surveyed in rural... This is important since a percentage is always relative to the n. 

 Did you assess understanding? Awareness and understanding are quite different and have an influence on understanding perceptions. 

 I hope you explain the discrepancy between low awareness and high acceptance in the discussions section; what could acceptance in this case mean?

 I see that all your tables and figures are located within the discussions section. Normally they would need to be in the results section, which makes it easier to refer to. Please correct accordingly, unless it is a journal requirement. 

**Discussion**

*Table 3*

 So, what is the difference between seeing/hearing info and awareness? If one hears information about GMM, does that not make them aware?

 Also, the total number aware of GMMs generally is 94, but then further down the table, it indicates that more people are aware of GMM work going on in Mali or of GMM-related legislation. This does not make any sense and is quite contradictory. Please revisit your analyses accordingly.

*Tables 4 & 5*

 Similar to table 3, this whole table is also confusing. Only 10% of your participants reported awareness of GMMs. When assessing perceptions, did you ask all participants, or just the 10% who were aware of GMMs? If you ask all, how do you determine that they knew what would be benefits or risks of what they were not aware of in the first place? I would suggest you revisit your analyses and include findings of only the 10% who were aware and see what their perceptions were. Otherwise, this information does not really make sense. Same with tables 5 and figure 2 

*Table 6*

 Please simplify this table to include only the most important info, and you can compress some of it. Otherwise, it is quite dense and hard to follow. I think you can simplify it to fit in one page

 I expected to see more critical discussion on what acceptability means when awareness is too low? 

 Also, more critical discussions on what acceptability means when there is no clear product represented. Which GMM technology would be more acceptable than the other? As they all have different implications.

 Also, you need a paragraph on study limitations, as there are quite a number of generalizations that were made, which is okay, but you need to acknowledge them. 

**Conclusion**

 The conclusion should stick to the study findings. In this study no info on community knowledge was assessed, and therefore the authors should not make conclusions on that. Also, authors should recommend next steps based on findings from this study.

Is the work clearly and accurately presented and does it cite the current literature?

Yes

If applicable, is the statistical analysis and its interpretation appropriate?

Partly

Are all the source data underlying the results available to ensure full reproducibility?

Yes

Is the study design appropriate and is the work technically sound?

Yes

Are the conclusions drawn adequately supported by the results?

Partly

Are sufficient details of methods and analysis provided to allow replication by others?

Partly

Reviewer Expertise:

Malaria, vector control, biocontrol technologies, community & stakeholder engagement

I confirm that I have read this submission and believe that I have an appropriate level of expertise to confirm that it is of an acceptable scientific standard, however I have significant reservations, as outlined above.
